# Supplementary material for: Astaxanthin Alleviates Foam Cell Formation and Promotes Cholesterol Efflux in Ox-LDL-Induced RAW264.7 Cells via CircTPP2/miR-3073b-5p/ABCA1 Pathway
Source: Molecules. 2023 Feb 10;28(4):1701. doi: 10.3390/molecules28041701 (PMC9961242; doi:10.3390/molecules28041701)
Supplement: Supplementary file 1 [file molecules-28-01701-s001.zip › molecules-2151618-supplementary.pdf]

Supplementary materials

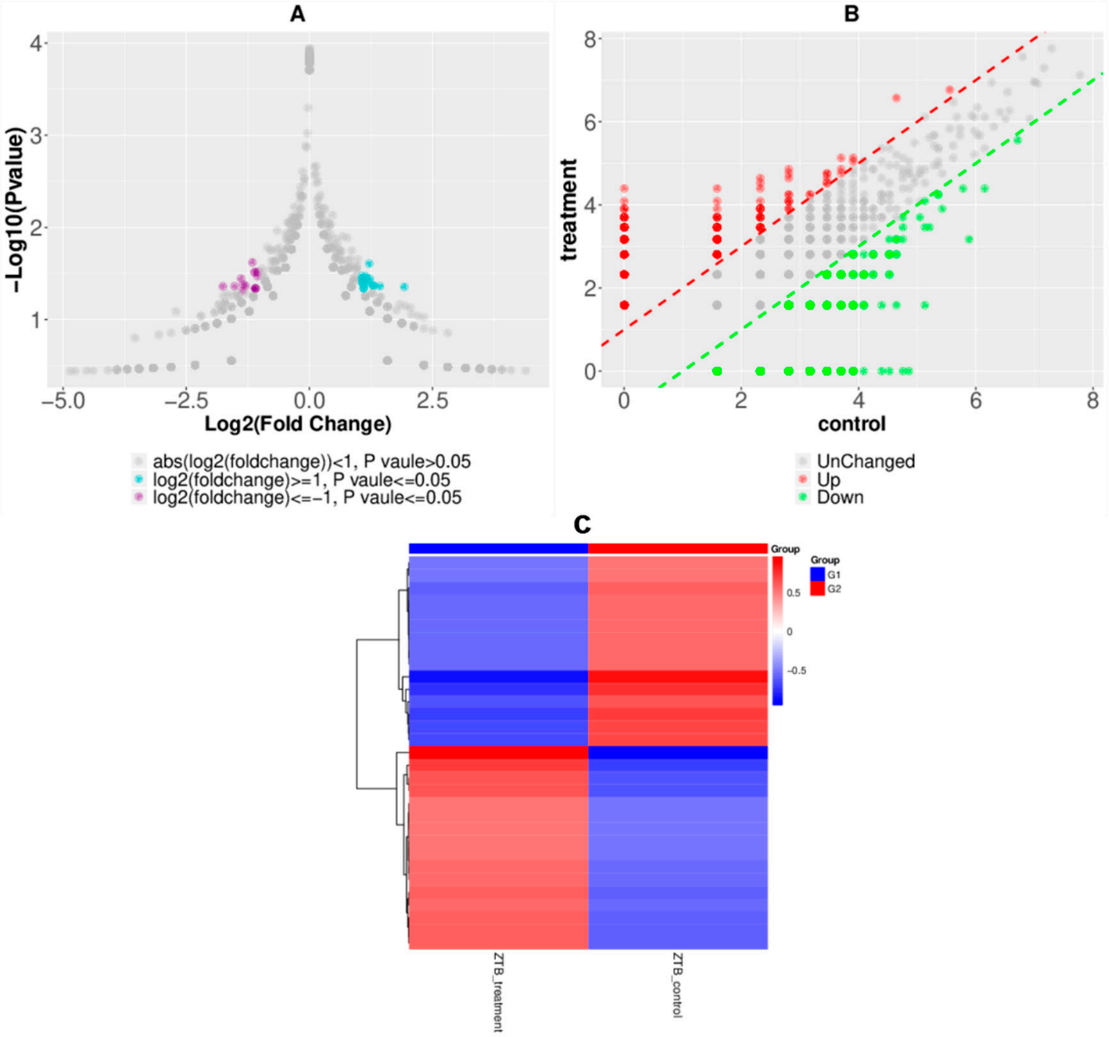

Figure S1. CircRNAs differentially expression pattern of the treat and control groups. The significant differentially expressed circRNAs between the two groups were illustrated in the Volcano plot (A) and the Scatter plot (B). The volcano plot showed the fold changes and p-values of circRNAs. The green and red blots of the scatter plot mean the significant DE-circRNAs. The black blots mean the non-significant DE-circRNAs. In the heat map (C), the color scale reflects the log2 signal intensity and runs from blue (low intensity), to white (medium intensity), to red (strong intensity). Up-regulated circRNAs are shown in red, and down-regulated circRNAs are shown in blue.

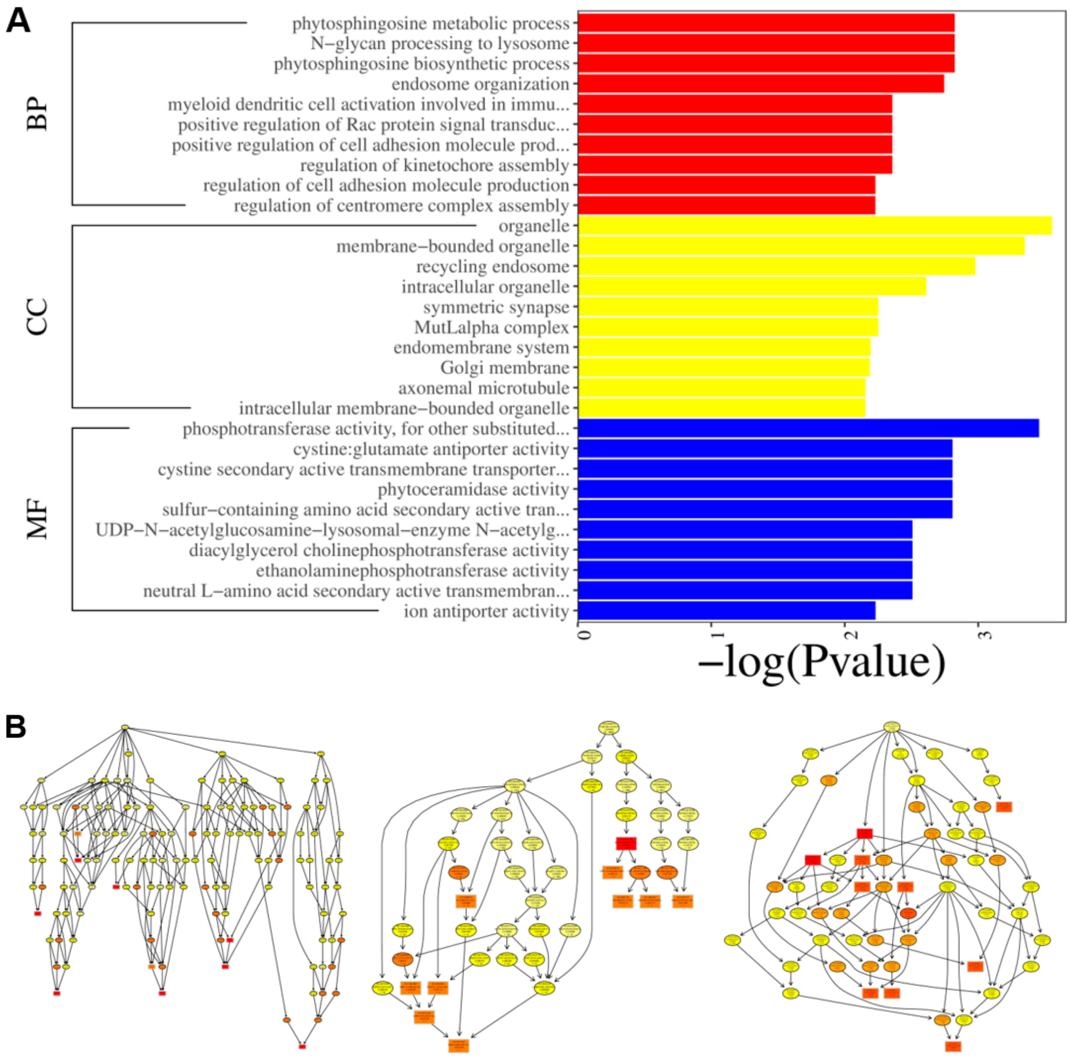

Figure S2. Significantly enriched GO histogram and dendrogram. GO analysis providing information concerning significantly enriched functions and the corresponding differentially expressed circRNAs covering three domains: biological process (BP), cellular component (CC) and molecular function (MF). According to the P-value ≤ 0.05 to filter the significant accumulation GO (A), the top 10 GOs are displayed under each GO category. The dendrogram (B) is CC, MF, BP from left to right, and the depth of the color indicates the degree of enrichment. The deeper the color, the higher the degree of enrichment.

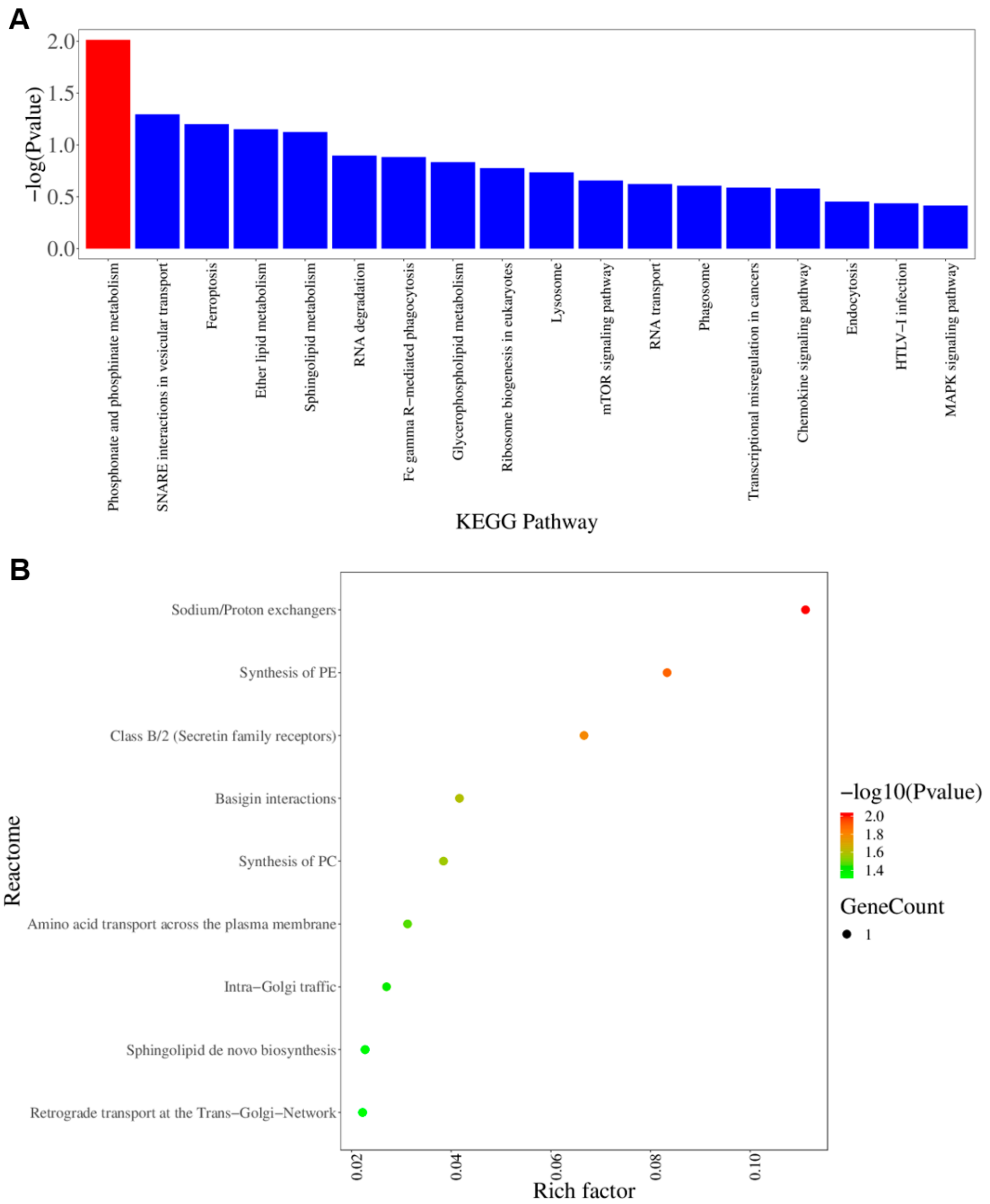

Figure S3. KEGG pathway histogram and scatter plot. (A) The histogram and (B) scatter plot shows the KEGG enriched analysis of circRNA-miRNA-mRNAs network of differentially expressed circRNA. The top 9 significantly enriched pathway and their scores (negative logarithm of P value) were listed as the x-axis and the y-axis, respectively.
